# Supplementary material for: Splice-Junction-Based Mapping of Alternative Isoforms in the Human Proteome
Source: Cell Rep. Author manuscript; Available in PMC 2020 Jan 15. (PMC6961840; doi:10.1016/j.celrep.2019.11.026)
Supplement: 2 [file NIHMS1546469-supplement-2.zip › DF1/PXD006675/LeftAtrium/LeftAtrium_11_TTN_SNCTVSVHVSEPPR.pdf]

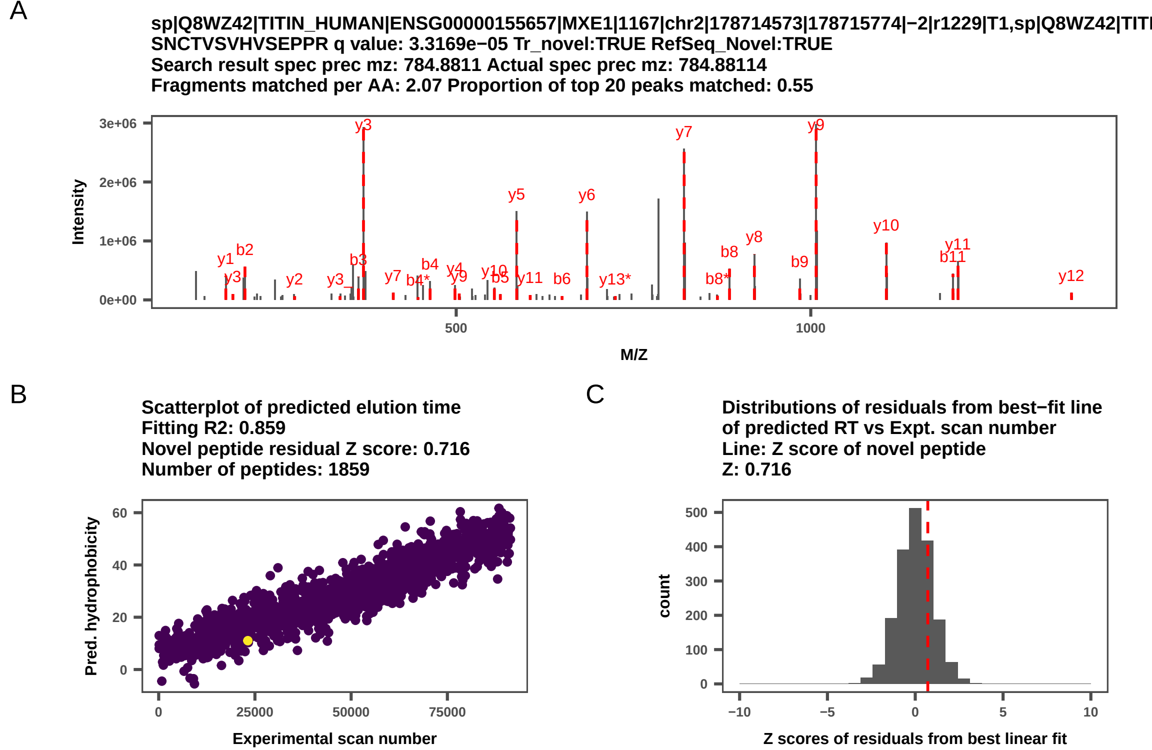
A
sp|Q8WZ42|TITIN_HUMAN|ENSG00000155657|MXE1|1167|chr2|178714573|178715774|−2|r1229|T1,sp|Q8WZ42|TITI
SNCTVSVHVSEPPR q value: 3.3169e−05 Tr_novel:TRUE RefSeq_Novel:TRUE
Search result spec prec mz: 784.8811 Actual spec prec mz: 784.88114
Fragments matched per AA: 2.07 Proportion of top 20 peaks matched: 0.55
Intensity
3e+06
2e+06
1e+06
0e+00
M/Z
500
1000
y1 b2 y3 y2 y3 b3 y3 y7 b4* b4 y4 y9 y10 b5 y5 y11 b6 y6 y13* y7 b8* b8 y8 b9 y9 y10 y11 b11 y12
B
Scatterplot of predicted elution time
Fitting R2: 0.859
Novel peptide residual Z score: 0.716
Number of peptides: 1859
Pred. hydrophobicity
60
40
20
0
0
25000
50000
75000
Experimental scan number
C
Distributions of residuals from best−fit line
of predicted RT vs Expt. scan number
Line: Z score of novel peptide
Z: 0.716
count
500
400
300
200
100
0
−10
−5
0
5
10
Z scores of residuals from best linear fit
